# Development and validation of a prognostic nomogram for predicting of patients with acute sedative-hypnotic overdose admitted to the intensive care unit

**DOI:** 10.1038/s41598-025-85559-1

**Published:** 2025-01-27

**Authors:** Guo Tang, Tianshan Zhang, Ping Zhang, Sha Yang, Tao Cheng, Rong Yao

**Affiliations:** Emergency Medicine Laboratory and the Department of Emergency, West China Hospital, Sichuan University, No. 37 Guoxue Alley, Chengdu, 610041 Sichuan China

**Keywords:** Acute Sedative-hypnotic overdose, Intensive care unit, Prognosis, Risk factors, Nomogram, Medical research, Risk factors, Human behaviour, Psychiatric disorders

## Abstract

To develop and evaluate a predictive model for intensive care unit (ICU) admission among patients with acute sedative-hypnotic overdose. We conducted a retrospective analysis of patients admitted to the emergency department of West China Hospital, Sichuan University, between October 11, 2009, and December 31, 2023. Patients were divided into ICU and non-ICU groups based on admission criteria including the need for blood purification therapy, organ support therapy (ventilatory support, vasoactive drugs, renal replacement therapy, artificial liver), or post-cardiopulmonary resuscitation. Patients were randomly split into a training set and a validation set in a 7:3 ratio. Least Absolute Shrinkage and Selection Operator (LASSO) regression was used to optimize variables, followed by a multivariate logistic regression analysis to identify independent risk factors for ICU admission. A nomogram model was constructed and assessed using receiver operating characteristic (ROC) curves, calibration curves, Decision Curve Analysis (DCA), and Clinical Impact Curve (CIC). Predictors in the nomogram included barbiturate overdose, Glasgow Coma Scale (GCS) score, and anion gap at admission. The nomogram demonstrated strong predictive performance with an area under the curve (AUC) of 0.858 (95% CI: 0.788–0.927) in the training set and 0.845 (95% CI: 0.757–0.933) in the validation set. Calibration curves showed the model closely matched the ideal curve, and DCA and CIC indicated high clinical applicability and utility. Barbiturate overdose, initial decreased GCS score and decreased anion gap were identified as independent risk factors for ICU admission in acute sedative-hypnotic overdose. The nomogram model based on these indicators demonstrates good predictive accuracy, discrimination, and clinical utility.

## Introduction

Acute drug overdose is the leading cause of acute poisoning globally and is a common condition encountered in emergency departments. This phenomenon is typically the result of drug overuse or inappropriate medication practices, with sedative-hypnotic overdoses being the most prevalent^1–8^. These substances primarily exert their effects by inhibiting gamma-aminobutyric acid (GABA) receptors in the brain, resulting in a series of complex psychological and physiological responses that often require urgent medical intervention^9–11^. Some patients may suffer severe organ damage or even death^12^. Reports indicate that the proportion of acute sedative-hypnotic overdoses admitted to intensive care unit (ICU) is the highest among all drug overdoses, ranging from 14.3 to 31.0%, with an in-hospital mortality rate of 1.0–3.6%^13,14^. Consequently, early identification of patients with acute sedative-hypnotic overdoses who require ICU admission could potentially enhance their prognosis. However, current clinical research on ICU admissions related to acute sedative-hypnotic overdoses is relatively scarce. This study aims to conduct a retrospective analysis of patients with acute sedative overdoses to identify associated risk factors for ICU admission and to develop a risk prediction model to inform early clinical intervention.

## Methods

### Study design

This study was a single-center, retrospective, cross-sectional study aimed at constructing and evaluating a nomogram model for predicting ICU admission in patients with acute sedative-hypnotic overdose, based on the latest predictive model guidelines and artificial intelligence reporting standards^15^.

### Study population

The study involved patients who presented to the emergency department of West China Hospital, Sichuan University, between October 10, 2009, and December 31, 2023, due to acute sedative-hypnotic overdose. The diagnosis of sedative-hypnotic overdose was established based on a clear history of exposure and corresponding clinical manifestations, including changes in mental status, general weakness, and abdominal pain, while excluding other diseases that present with similar clinical features^14^. According to the International Classification of Diseases, 10th Revision (ICD-10), sedative-hypnotics were classified into three categories: barbiturates, benzodiazepines, and Z-drugs^16^.

The inclusion criteria for the study were: (1) age ≥ 14 years; and (2) onset of symptoms within 72 h. The exclusion criteria included: (1) co-occurrence with other types of poisoning (e.g., alcohol, pesticides); (2) co-occurrence with acute physical or chemical injuries (e.g., burns, trauma); and (3) incomplete clinical information.

### Variables and grouping

The study variables encompassed general information, including age, sex, the presence of psychiatric disorders along with their specific diagnoses, and the types of drugs ingested. In cases of mixed drug overdose, more than two classes of drugs were involved; if patients or their families reported an overdose of sedative-hypnotic agents but could not specify the exact drug, these were categorized as unspecified drugs. Additional variables included the time from overdose to presentation, as well as clinical signs at presentation such as temperature, heart rate, respiratory rate, peripheral oxygen saturation, systolic and diastolic blood pressure, and Glasgow Coma Scale (GCS) scores. Initial laboratory results measured included hemoglobin, platelet count, white blood cell count, absolute neutrophil count, absolute lymphocyte count, total bilirubin, direct bilirubin, alanine aminotransferase, aspartate aminotransferase, albumin, glucose, urea, creatinine, uric acid, sodium, potassium, chloride, and anion gap. In-hospital treatment interventions included toxic substance removal, administration of specific antidotes, organ support therapy, and cardiopulmonary resuscitation.

Patients were categorized into an ICU admission group and a non-ICU admission group based on their admission status to the ICU during their hospital stay. Indications for ICU admission included the need for blood purification therapy, organ support therapy (such as mechanical ventilation, vasoactive drugs, renal replacement therapy, or artificial liver support), or post-cardiopulmonary resuscitation care. Disease severity, clinical signs at presentation, and initial laboratory results were compared between the two groups.

All patients were monitored until discharge, with the endpoint defined as the discharge status, which was categorized as improved or recovered; not improved or worsened; or deceased.

### Sample size calculation

The sample size was calculated using the “pmsampsize” function to ensure adequate statistical power^17^. Parameters included a C-index of 0.8, 4 candidate predictors, an overall event rate of 0.14, resulting in a required sample size of 231, with 8.09 events per candidate predictor.

### Statistical analysis

Statistical analysis was performed using R 4.4.1 software. Normally distributed continuous variables were presented as mean ± standard deviation (x ± s) and compared between groups using the t-test. Skewed continuous variables were presented as median (interquartile range) and compared using the Mann-Whitney U test. Categorical variables were presented as n (%) and compared using the chi-square test or Fisher’s exact test.

Patients were randomly divided into training and validation sets in a 7:3 ratio through simple random sampling without replacement. In the training set, the least absolute shrinkage and selection operator (LASSO) regression was employed to optimize variables, thereby mitigating potential multicollinearity and overfitting. The selected variables were subsequently included in a multivariate logistic regression analysis utilizing a forward stepwise method to identify independent risk factors for ICU admission, and a nomogram model was constructed. The model’s performance was assessed using receiver operating characteristic (ROC) curves, calibration curves, decision curve analysis (DCA), and clinical impact curves (CIC) in both the training and validation sets^18–21^. Internal validation was conducted through bootstrap resampling with 1000 resamples^22^.

## Results

### Features and grouping

**Fig. 1:**
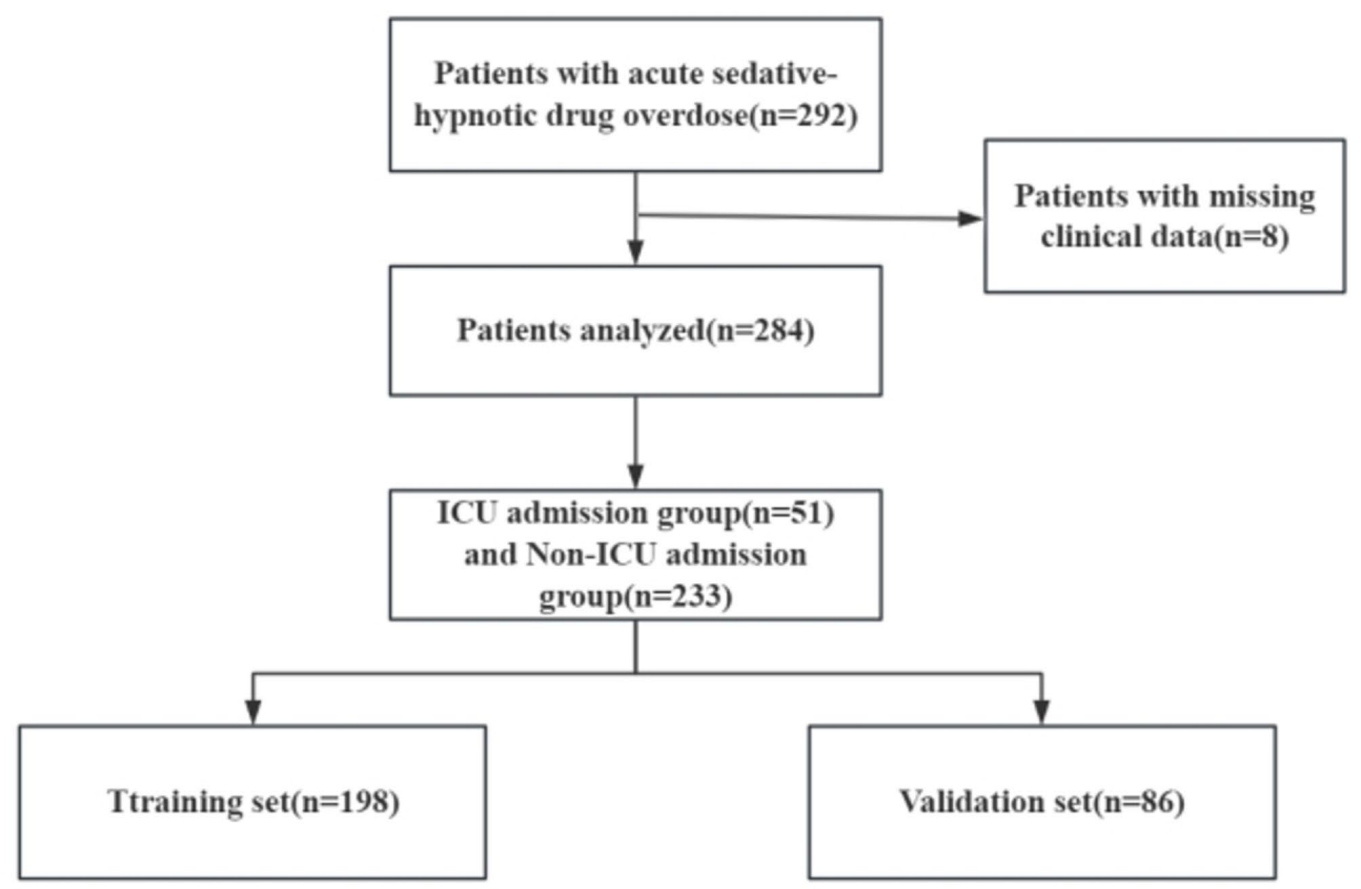
Flowchart of the study.

During the study period, a total of 292 patients with acute sedative-hypnotic overdose presented to the emergency department. Based on the established inclusion and exclusion criteria, 284 patients were included in the analysis (Fig. 1), of which 225 (80.3%) were female, with an average age of 42.0 years (range: 24.8 to 56.0 years). ICU admission was necessary for 51 (18.0%) of the patients. Compared to the non-ICU group, the ICU group exhibited a higher prevalence of a history of mental illness (66.7% vs. 48.5%, *P* = 0.028), a greater proportion of barbiturate use (9.8% vs. 1.29%, *P* = 0.006), higher white blood cell counts (7.54 × 10⁹/L vs. 6.58 × 10⁹/L, *P* = 0.002), absolute neutrophil counts (5.76 × 10⁹/L vs. 4.41 × 10⁹/L, *P* < 0.001), and glucose levels (5.99 mmol/L vs. 5.44 mmol/L, *P* = 0.001). Conversely, the ICU group demonstrated lower GCS scores (3 vs. 15, *P* < 0.001), hemoglobin levels (118 g/L vs. 128 g/L, *P* < 0.001), absolute lymphocyte counts (1.31 × 10⁹/L vs. 1.53 × 10⁹/L, *P* = 0.013), direct bilirubin levels (2.50 µmol/L vs. 3.40 µmol/L, *P* = 0.034), albumin levels (40.4 g/L vs. 42.5 g/L, *P* = 0.003), and anion gap values (11.1 µmol/L vs. 15.2 µmol/L, *P* < 0.001) (Table 1). Patients were randomly divided into training (198 patients) and validation (86 patients) sets in a 7:3 ratio. In the training set, 158 (79.8%) were female, with an average age of 37 years (25 to 56 years), and ICU admission was required for 30 (15.2%) patients. In the validation set, 67 (77.9%) were female, with an average age of 40.0 years (range: 24.0 to 54.5 years), and ICU admission was required for 21 (24.4%) patients. No statistically significant differences were found between the training and validation sets (Table 2).

**Table 1:** Comparison between ICU-admitted and Non-ICU-admitted patients.

Risk factors	Non-ICU admission group (*N* = 233)	ICU admission group(*N* = 51)	*P*
Sex (female)	181 (77.7%)	44 (86.3%)	0.238
Age (years)	37.0 [25.0;53.0]	46.0 [22.5;69.0]	0.184
Time from poisoning to treatment at the study site (h)	3.00 [2.00;10.0]	5.00 [2.00;14.0]	0.093
Presence of psychiatric history(%)	113 (48.5%)	34 (66.7%)	0.028
Depression(%)	74 (31.8%)	22 (43.1%)	0.164
Bipolar Disorder(%)	11 (4.72%)	5 (9.80%)	0.177
Schizophrenia(%)	6 (2.58%)	3 (5.88%)	0.207
Anxiety(%)	6 (2.58%)	2 (3.92%)	0.638
Other Psychiatric Disorders(%)	20 (8.58%)	3 (5.88%)	0.777
Types of overdose			
Benzodiazepines(%)	176 (75.5%)	43 (84.3%)	0.243
Barbiturates(%)	3 (1.29%)	5 (9.80%)	0.006
Z-Drugs(%)	19 (8.15%)	1 (1.96%)	0.141
Unknown(%)	3 (1.29%)	0 (0.00%)	1.000
Polydrug Overdose(%)	32 (13.7%)	2 (3.92%)	0.086
Clinical signs at presentation laboratory			
Temperature(℃)	36.5 [36.3;36.7]	36.5 [36.2;36.7]	0.948
Heart Rate (beat/min)	85.0 [73.0;97.0]	87.0 [73.5;99.0]	0.371
Respiratory Rate (beats/min)	20.0 [19.0;20.0]	20.0 [18.5;20.5]	0.859
Systolic blood pressure(mmHg)	115 [103;126]	112 [102;126]	0.679
Diastolic blood pressure(mmHg)	72.0 [65.0;80.0]	71.0 [64.5;80.5]	0.571
Peripheral Oxygen Saturation(%)	97.0 [96.0;99.0]	97.0 [96.0;98.0]	0.889
Glasgow Coma Scale	15.0 [11.0;15.0]	3.00 [3.00;11.5]	<0.001
Results at presentation			
Hemoglobin(g/L)	128 [120;137]	118 [109;128]	<0.001
Platelet Count(×10^9^/L)	197 [154;238]	207 [150;244]	0.772
White Blood Cell Count (×10^9^/L)	6.58 [5.34;8.06]	7.54 [6.42;10.3]	0.002
Absolute Neutrophil Count (10^9/L)	4.41 [3.16;6.00]	5.76 [4.39;8.19]	<0.001
Absolute Lymphocyte Count (10^9/L)	1.53 [1.19;1.92]	1.31 [0.86;1.70]	0.013
Total Bilirubin(µmol/l)	10.3 [7.40;13.6]	8.60 [6.00;13.3]	0.155
Direct Bilirubin(µmol/l)	3.40 [2.30;4.70]	2.50 [1.80;4.30]	0.034
Alanine Aminotransferase (IU/L)	14.0 [10.0;22.0]	13.0 [8.50;19.5]	0.177
Aminotransferase (IU/L)	19.0 [15.0;26.0]	17.0 [14.0;24.5]	0.572
Albumin (g/L)	42.5 [40.2;44.8]	40.4 [37.7;43.3]	0.003
Glucose(mmol/L)	5.44 [4.83;6.10]	5.99 [5.39;7.54]	0.001
Blood Urea Nitrogen (mmol/L)	4.20 [3.30;5.31]	3.70 [3.02;4.75]	0.139
Serum Creatinine(µmol/L)	61.0 [52.0;71.0]	60.0 [54.0;67.5]	0.791
Uric Acid(mmol/L)	286 [228;345]	274 [196;338]	0.162
Blood Sodium (mmol/L)	139 [137;141]	138 [136;139]	0.063
Blood Potassium (mmol/L)	3.59 [3.35;3.78]	3.51 [3.21;3.76]	0.156
Chloride (mmol/L)	104 [102;107]	104 [102;107]	0.513
Anion Gap(umol/L)	15.2 [11.9;17.7]	11.1 [8.25;15.4]	<0.001
Clinical outcomes			<0.001
Cured (%)	232 (99.6%)	44 (86.3%)	
Failure to recover or automatic discharge (%)	1 (0.43%)	6 (11.8%)	
Deaths (%)	0 (0.00%)	1 (1.96%)	

**Table 2:** Comparison of baseline characteristics between the training and validation sets.

Risk factors	Training set (*N* = 198)	Validation set (*N* = 86)	*P*
Sex (female)	158 (79.8%)	67 (77.9%)	0.840
Age (years)	37.0 [25.0;56.0]	40.0 [24.0;54.5]	0.706
Time from poisoning to treatment at the study site (h)	3.00 [2.00;10.0]	4.00 [2.00;10.8]	0.235
Presence of psychiatric history (%)	104 (52.5%)	43 (50.0%)	0.793
Depression (%)	71 (35.9%)	25 (29.1%)	0.330
Bipolar disorder (%)	10 (5.05%)	6 (6.98%)	0.578
Schizophrenia (%)	5 (2.53%)	4 (4.65%)	0.461
Anxiety (%)	4 (2.02%)	4 (4.65%)	0.250
Other psychiatric disorders (%)	17 (8.59%)	6 (6.98%)	0.826
Types of overdose			
Benzodiazepines (%)	154 (77.8%)	65 (75.6%)	0.802
Barbiturates (%)	6 (3.03%)	2 (2.33%)	1.000
Z-Drugs (%)	11 (5.56%)	9 (10.5%)	0.217
Unknown (%)	25 (12.6%)	9 (10.5%)	0.752
Polydrug overdose (%)	2 (1.01%)	1 (1.16%)	1.000
Clinical signs at presentation laboratory			
Temperature (℃)	36.5 [36.2;36.7]	36.5 [36.3;36.7]	0.598
Heart Rate (beat/min)	85.0 [73.0;97.0]	85.0 [72.2;96.0]	0.875
Respiratory rate (beats/min)	20.0 [19.0;20.0]	20.0 [19.0;21.0]	0.318
Systolic blood pressure (mmHg)	115 [103;127]	113 [103;125]	0.747
Diastolic blood pressure (mmHg)	72.0 [66.0;80.0]	70.5 [64.2;79.0]	0.179
Peripheral oxygen saturation (%)	97.0 [95.2;98.0]	97.0 [96.0;98.0]	0.722
Glasgow coma scale	14.0 [9.00;15.0]	15.0 [5.75;15.0]	0.862
Results at presentation			
Hemoglobin (g/L)	127 [118;136]	126 [116;136]	0.739
Platelet count (×10^9^/L)	196 [154;243]	202 [150;234]	0.998
White blood cell count (×10^9^/L)	6.64 [5.39;8.53]	6.96 [5.82;8.33]	0.541
Absolute neutrophil count (×10^9^/L)	4.54 [3.24;6.31]	4.77 [3.46;6.27]	0.652
Absolute lymphocyte count (×10^9^/L)	1.48 [1.10;1.89]	1.52 [1.09;1.90]	0.848
Total bilirubin (µmol/l)	10.4 [6.93;13.6]	9.00 [6.82;13.4]	0.687
Direct bilirubin (µmol/l)	3.30 [2.02;4.50]	3.30 [2.30;4.77]	0.664
Alanine aminotransferase (IU/L)	14.0 [10.0;22.0]	14.0 [9.25;20.0]	0.752
Aminotransferase (IU/L)	19.0 [15.0;26.0]	17.5 [15.0;22.0]	0.332
Albumin (g/L)	42.3 [39.7;44.5]	42.0 [39.5;44.8]	0.767
Glucose (mmol/L)	5.55 [5.04;6.50]	5.14 [4.80;6.10]	0.027
Blood urea nitrogen (mmol/L)	4.20 [3.20;5.27]	4.02 [3.24;5.07]	0.987
Serum creatinine (µmol/L)	61.0 [52.0;70.0]	60.0 [52.0;69.8]	0.542
Uric acid (mmol/L)	286 [222;342]	276 [211;364]	0.783
Blood sodium (mmol/L)	139 [137;140]	139 [137;141]	0.275
Blood potassium (mmol/L)	3.59 [3.37;3.78]	3.56 [3.26;3.79]	0.406
Chloride (mmol/L)	104 [102;107]	104 [102;106]	0.680
Anion Gap (umol/L)	14.6 [10.9;17.7]	15.2 [12.5;17.4]	0.341

### Construction of the nomogram model

**Fig. 2:**
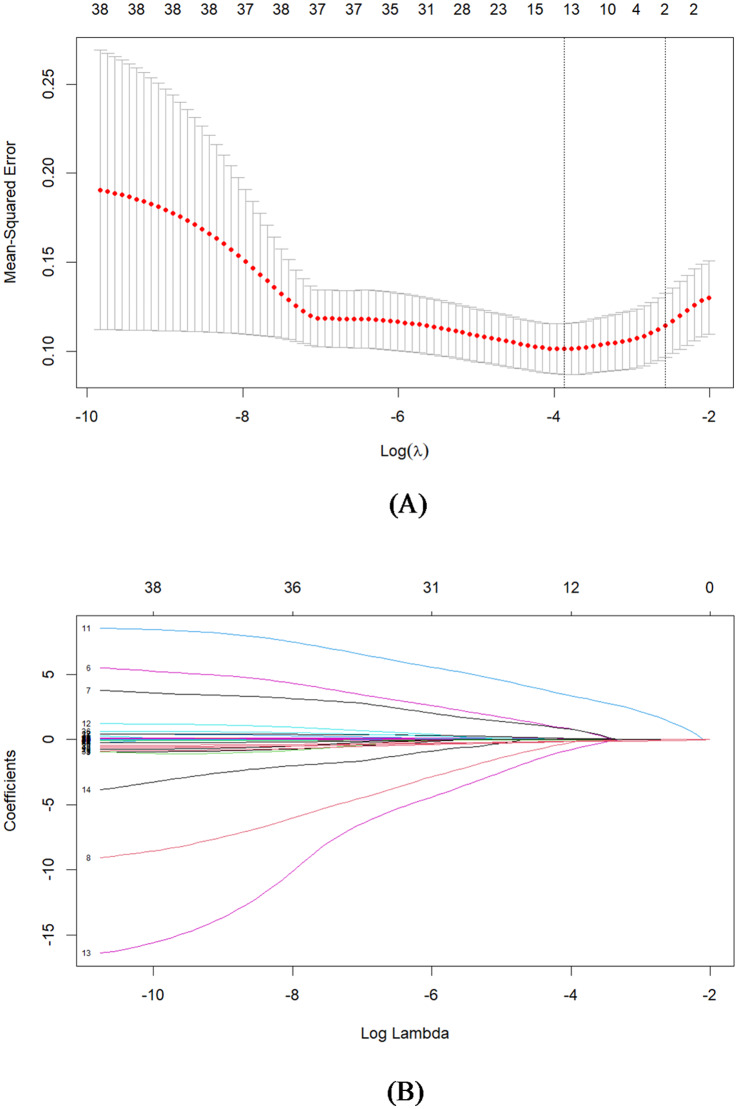
LASSO regression was used for demographic and clinical feature selection. (A) LASSO regression cross-validation results; (B) LASSO regression coefficient log (Lambda) profile.

**Fig. 3:**
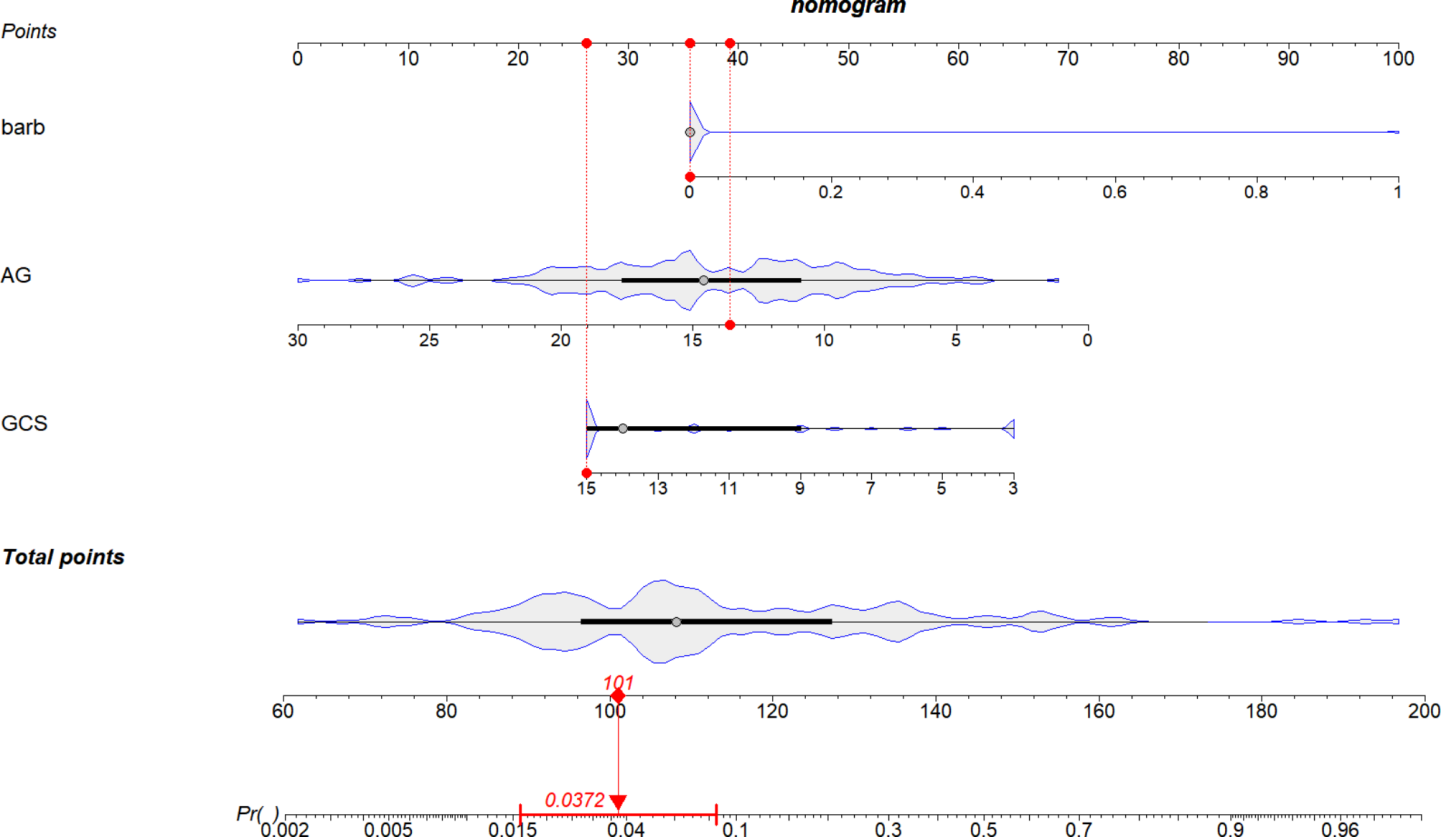
Nomogram model of patients with acute sedative-hypnotic drug overdose admitted to ICU.

In this study, demographic and clinical data from 198 patients with sedative-hypnotic overdose were analyzed. The “glmnet” package was utilized to perform LASSO regression, which facilitated the selection of feature variables (Fig. 2). This analysis identified three significant variables: the presence of barbiturate overdose, the GCS score at presentation, and the anion gap. These variables were subsequently incorporated into a multivariate logistic regression analysis using a forward stepwise method to ascertain independent risk factors. The results indicated that barbiturate overdose (OR: 111.62, 95% CI: 9.09-1370.5), a decreased GCS score at presentation (OR: 0.79, 95% CI: 0.71–0.87), and a reduced anion gap (OR: 0.84, 95% CI: 0.75–0.94) were independent predictors of ICU admission (Table 3). Additionally, a nomogram was constructed based on these three indicators (Fig. 3), where each predictor was assigned a point on the nomogram, and the total score corresponded to the probability of ICU admission, displayed at the bottom.

**Table 3:** Risk factors of patients with acute sedative-hypnotic drug overdose admitted to ICU.

Variable	Crude OR (95% CI)	Adjusted OR (95% CI)	*P*(Wald’s test)	*P*(LR test)
Barbiturates	33.4(3.75,297.78)	111.62(9.09,1370.5)	< 0.001	< 0.001
Glasgow Coma Scale	0.81 (0.75,0.89)	0.79 (0.71,0.87)	< 0.001	< 0.001
Anion Gap	0.87 (0.8,0.96)	0.84 (0.75,0.94)	0.002	< 0.001

### Evaluation of the nomogram model

**Fig. 4:**
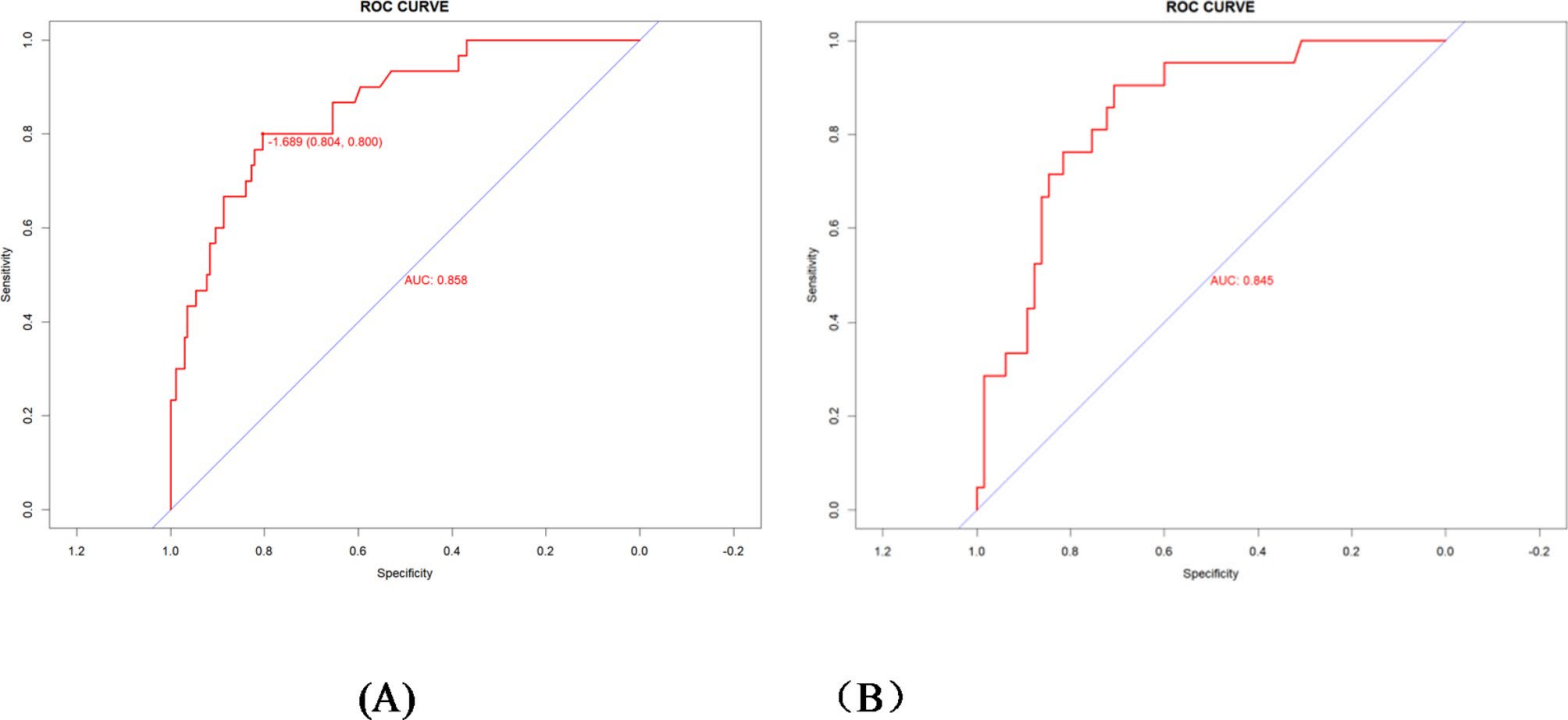
(**A**) ROC curve of the nomogram in the training set; (**B**) ROC curves of the nomogram in the validation set. Notes: ROC: Receiver Operating Characteristic Curve; AUC: area under the curve.

**Fig. 5:**
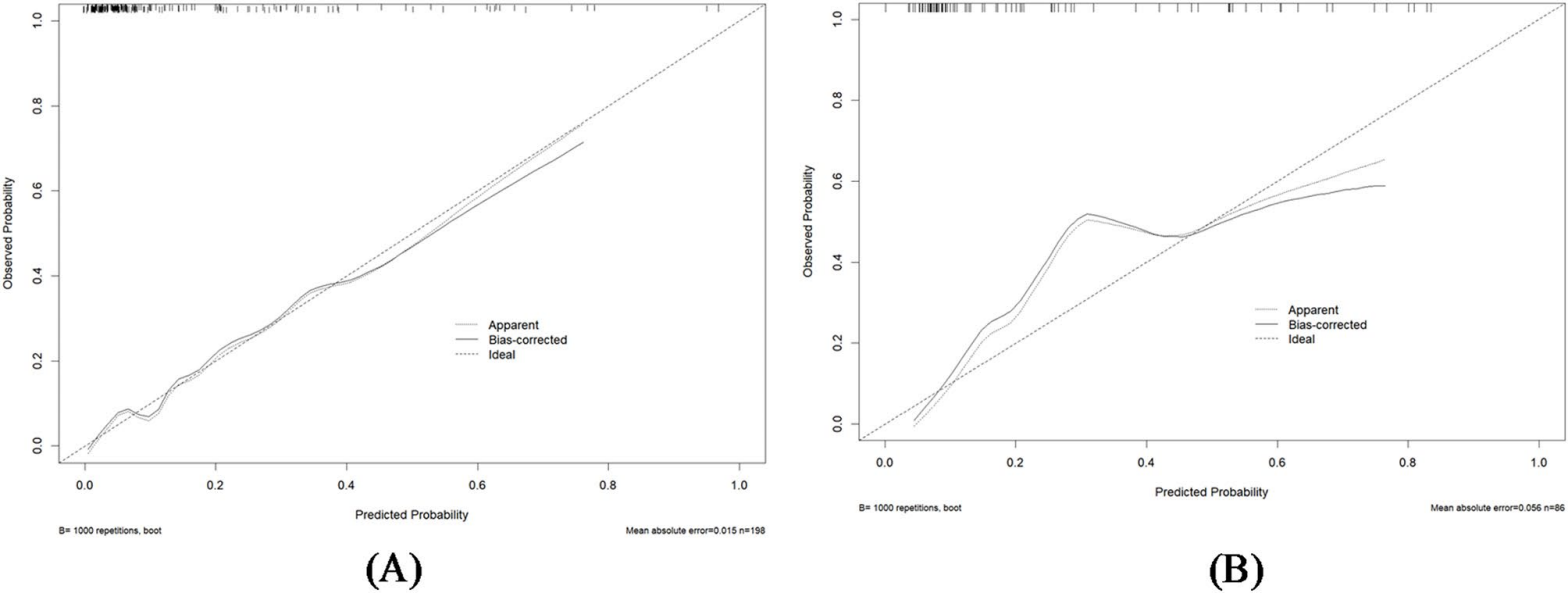
(**A**) Calibration curve of the nomogram in the training set; (**B**) Calibration curves of the nomogram in the validation set.

**Fig. 6:**
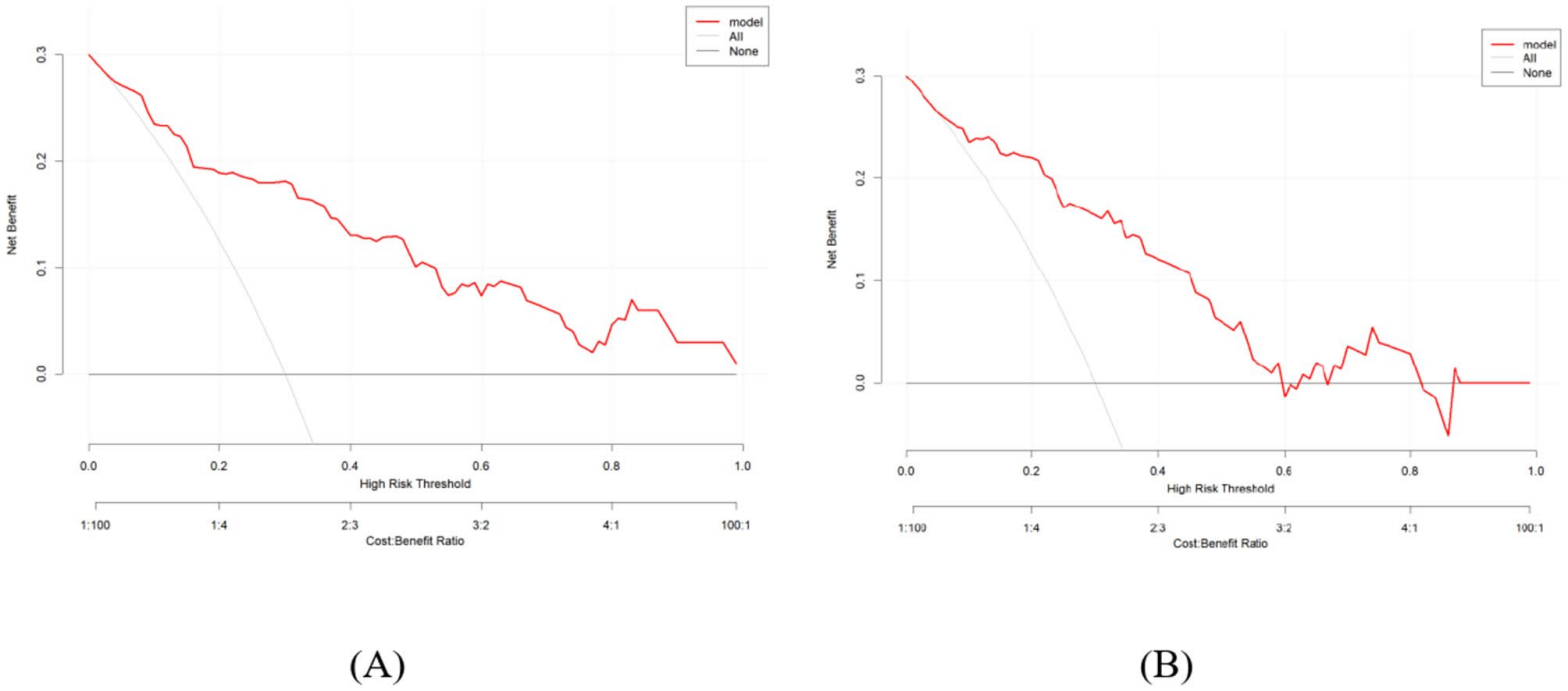
(**A**) Decision curve analysis of the nomogram in the training set; (**B**) Decision curve analysis of the nomogram in the validation set.

**Fig. 7:**
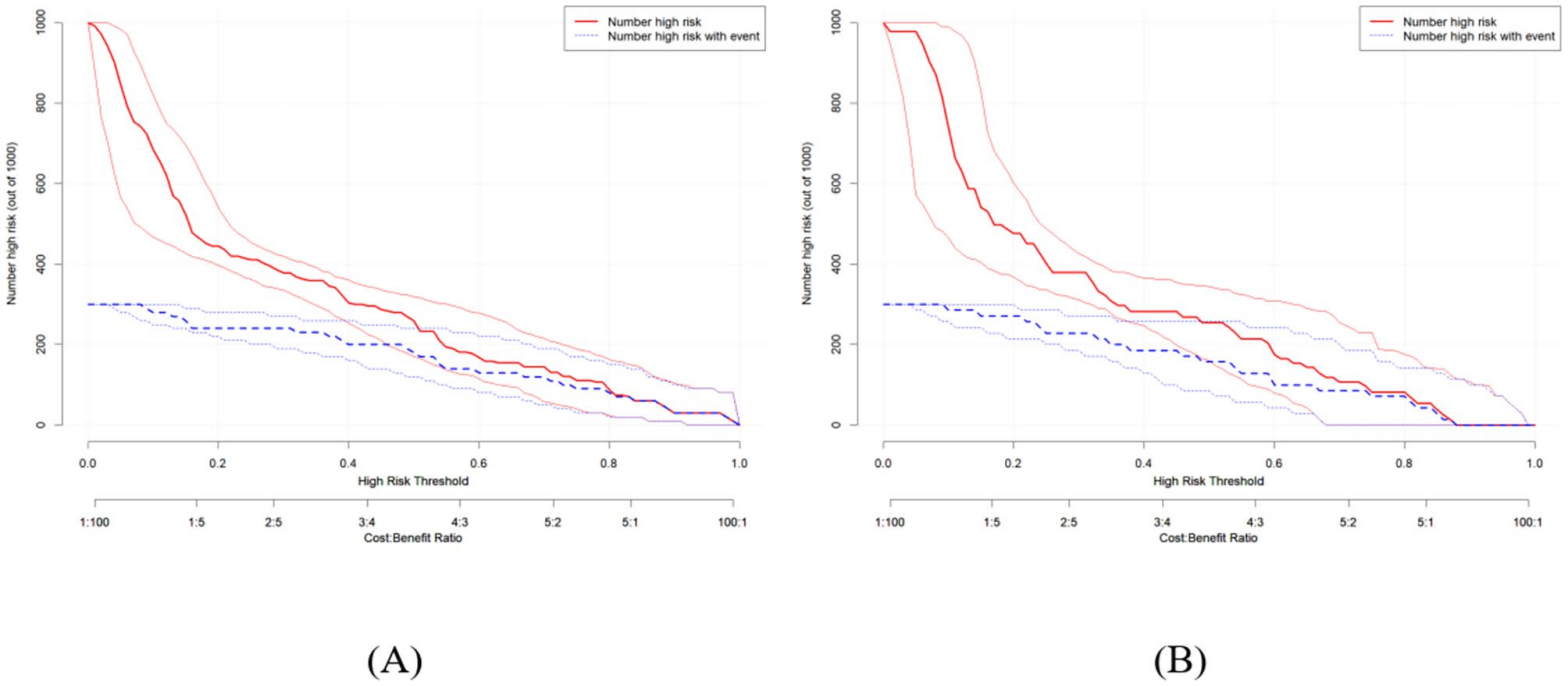
(**A**) Clinical Impact Curve of the nomogram in the training set; (**B**) Clinical Impact Curve of the nomogram in the validation set.

To evaluate the performance of the predictive model, we plotted ROC curves, calibration curves, DCA curves, and CIC curves for both the training and validation sets. Figure 4(A) and 4(B) present the ROC curves for these sets, demonstrating that the model achieved an AUC of 0.858 (95% CI: 0.788–0.927) in the training set and 0.845 (95% CI: 0.757–0.933) in the validation set. Furthermore, the calibration curves illustrated strong consistency between predicted and observed outcomes in both sets (Fig. 5A and B). The DCA curves revealed a significant net benefit of the predictive model (Fig. 6A and B), while the CIC curves indicated substantial clinical utility with thresholds exceeding 0.4 in both sets (Fig. 7A, B).

## Discussion

Sedative-hypnotic overdose represents an increasingly serious public health concern. Patients frequently present with transient disturbances in consciousness; while most individuals show improvement with prompt treatment, a subset may progress to severe illness, resulting in significant organ damage or even death^12^. This study found that approximately 18% of patients required admission to the ICU. Previous studies have demonstrated significant variability in the probability of drug overdose patients requiring ICU admission, with rates ranging from 1.3–36.4%^14,23–31^. Cioccari et al. analyzed 18,050 drug overdose cases and reported an ICU admission rate of 1.3%^29^. In contrast, Sharif et al. studied 143 patients poisoned by substances with central nervous system toxicity and found an ICU admission rate of 36.4%^14^. This discrepancy may be attributed to differences in the types of drugs involved and variations in ICU admission criteria. In this study, patients admitted to the ICU demonstrated significantly lower recovery rates and higher in-hospital mortality rates compared to their non-ICU counterparts. Consequently, a prompt and accurate assessment of disease severity is essential for formulating appropriate treatment plans and enhancing patient outcomes. However, there is currently a deficiency of specific tools for evaluating the severity of acute sedative-hypnotic overdose in emergency settings. Through clinical data analysis, this study identified three independent risk factors for ICU admission in patients with acute sedative-hypnotic overdose: the presence of barbiturate overdose, a decreased GCS score and anion gap at presentation. A nomogram model was constructed incorporating these factors, demonstrating strong predictive performance with an area under the curve (AUC) of 0.858 (95% CI: 0.788–0.927) in the training set and an AUC of 0.845 (95% CI: 0.757–0.933) in the validation set. The model exhibited good clinical applicability and practicality, as indicated by calibration curves, DCA curves, and CIC. Since these indicators are readily obtainable shortly after patient presentation, the model is feasible for emergency application and dissemination, potentially providing clinicians with more accurate early assessments of disease severity and prognosis.

This study identifies a decreased GCS score at presentation as a risk factor for ICU admission in patients experiencing acute sedative-hypnotic overdose. Altered consciousness is a prevalent characteristic of drug overdose^32^. The underlying mechanisms may involve the effects of these substances on various neurotransmitter receptors within the brain. Existing research indicates that GABA receptors are the primary targets for benzodiazepines and barbiturates, which enhance GABAergic transmission and produce a pharmacological effect^9^. GABA receptors serve as the main inhibitory neurotransmitter receptors in the central nervous system, regulating chloride ion channels, activating G proteins, causing cell hyperpolarization, slowing impulse transmission, or inhibiting adenylate cyclase activity, thereby resulting in an overall inhibitory effect^10,11,33–36^. Changes in the level of consciousness are associated with the dosage or type of ingested drugs, which can increase the risk of complications such as respiratory depression and aspiration, making it a valuable indicator of the severity of antipsychotic overdose. Additionally, our study identifies the anion gap as an independent risk factor for ICU admission in patients with acute sedative-hypnotic overdose, consistent with previous reports^37–40^. The anion gap is a laboratory measurement that reflects the difference between serum cations and anions, indicating the concentration of unmeasured anions. In cases of metabolic acidosis caused by the accumulation of strong acids, such as lactate or beta-hydroxybutyrate in extracellular fluid, bicarbonate levels decrease while the anion gap increases. The extent of the anion gap elevation correlates with the plasma concentration of these accumulated anions. Potential causes of an increased anion gap include ingestion of ethylene glycol, acetaminophen, methanol, aspirin, renal failure, or ketoacidosis^39^. Furthermore, existing literature suggests that a reduction in the anion gap may indicate the presence of electrolyte metabolism disorders or hypoalbuminemia in the patient^40^. In patients with acute sedative-hypnotic overdose, decreased anion gaps may be the result of homeostatic imbalance related to multiple mechanisms^41,42^.

Barbiturate overdose constitutes an independent risk factor for ICU admission in patients experiencing acute sedative-hypnotic overdose. Research on phenobarbital overdose is limited, primarily consisting of case reports^43–45^. Satoshi et al. conducted a retrospective study involving 277 patients who presented with drug overdoses at a hospital in Tokyo from 2011 to 2012^46^. They found that barbiturate overdose is an independent risk factor for the development of aspiration pneumonia and the need for endotracheal intubation. Existing literature indicates that barbiturates predominantly impact the central nervous system, potentially leading to shallow breathing and coma in severe cases^47^. The concomitant use of barbiturates with other central nervous system depressants, such as benzodiazepines, exacerbates the depressant effects on both the central nervous and respiratory systems, making overdose particularly hazardous^47^. Therefore, recognizing barbiturate use in cases of sedative-hypnotic overdose is crucial for facilitating early intervention and treatment. In patients with acute sedative-hypnotic overdose, a decreased anion gap may arise from homeostatic imbalances related to multiple mechanisms.

Few studies have focused on ICU admissions related to sedative-hypnotic overdose. Lu et al. conducted a retrospective analysis of 140 cases of acute benzodiazepine overdose in patients aged 18 and older, treated in the emergency department of Taipei Veterans General Hospital between 2012 and 2015^13^. Their findings revealed that 97 patients (69.2%) required hospitalization, 20 patients (14.3%) were admitted to the ICU, and 3 patients (2.1%) succumbed to their condition. Multivariate analysis identified concomitant pneumonia and the administration of flumazenil in the emergency department as independent risk factors for ICU admission. However, the study’s small sample size limited its findings, and it did not develop a nomogram model for clinical prediction. Additionally, research on ICU admissions for barbiturate and Z-drug overdoses is limited, highlighting the need for further investigation in this area.

This study has several limitations. Firstly, it was a single-center, retrospective investigation that exclusively included clinical data obtained at the time of hospital admission, without follow-up on post-discharge survival, which may influence the results. Moreover, the vital signs recorded during visit were manually measured by medical staff, potentially leading some inaccuracies. Prospective studies are necessary to assess the long-term prognostic significance of these indicators and to implement follow-up monitoring of high-risk populations. Additionally, due to limitations in sample size, external validation of the predictive model was not conducted, highlighting the need for further evaluation of the model’s efficacy. In conclusion, while our research is comprehensive, the results may be influenced by advancements in blood purification treatment technologies, ongoing developments in liver and stomach protective medications, and improvements in antibiotic treatments.

## Conclusion

This study identified barbiturate overdose, a decreased GCS score at presentation, and a reduced anion gap as independent risk factors for ICU admission in patients experiencing acute sedative-hypnotic overdose. The nomogram model developed from these indicators exhibited strong predictive performance for ICU admission in this patient population.

## Data Availability

The datasets generated during and/or analyzed during the current study are not publicly available due to data confidentiality. However, they can be obtained from the corresponding author upon reasonable request.
